# Nanocomposites Based on Polyethylene and Nickel Ferrite: Preparation, Characterization, and Properties

**DOI:** 10.3390/polym15193988

**Published:** 2023-10-04

**Authors:** Gleb Yurkov, Alexander Kozinkin, Stanislav Kubrin, Alexander Zhukov, Svetlana Podsukhina, Valeriy Vlasenko, Alexander Fionov, Vladimir Kolesov, Dmitry Zvyagintsev, Maria Vyatkina, Vitaliy Solodilov

**Affiliations:** 1 N.N. Semenov Federal Research Center of Chemical Physics, Russian Academy of Sciences, Kosygina 4, 119991 Moscow, Russia; zhukov765311@yandex.ru (A.Z.); vital-yo@yandex.ru (V.S.); 2 Research Institute of Physics, Southern Federal University, pr. Stachki 194, 344090 Rostov-on-Don, Russia; 3 Kotelnikov Institute of Radio Engineering and Electronics of RAS, 125009 Moscow, Russia

**Keywords:** composites, nanoparticles, nickel ferrite, polyethylene, atomic structure, electronic structure, Mössbauer spectroscopy, X-ray diffraction, X-ray absorption spectroscopy, Fourier transform modulus, electrophysical and magnetic properties

## Abstract

Composite materials based on NiFe_2_O_4_ nanoparticles and polyethylene matrix have been synthesized by thermal decomposition to expand the application area of high-pressure polyethylene by filling it with nanoscale particles. The synthesized compositions were obtained in the form of a dark gray powder and compressed for further study According to TEM, the average particle size in composites was 2, 3, and 4 nm in samples with a filling of 10%, 20% and 30%. The concentration dependences of the specific electrical resistivity ρ_V_, dielectric permittivity ε, saturation magnetization M_S_ and the parameters of reflection and attenuation of microwave power of the obtained composites were investigated. The threshold for percolation in such materials is found to be within a concentration range of 20…30%. The electronic and atomic structure of composites was studied by methods of Mössbauer spectroscopy, X-ray diffraction and X-ray absorption spectroscopy. The closest atomic environment of nickel and iron in nanoparticles is close to that of crystalline NiFe_2_O_4_. The dependence of the nanoparticles size as well as the dependence of the number of tetrahedral or octahedral iron positions in nickel ferrite nanoparticles to their content in polyethylene matrix is established. It is shown that composite materials based on NiFe_2_O_4_ nanoparticles and polyethylene matrix can be used as components of electromagnetic compatibility systems.

## 1. Introduction

High-pressure polyethylene (HPPE) is the most exploited polymer, it is used in the production of parts of various devices, coatings and medical devices [1,2]. Expansion of HPPE sphere of application is possible due to creation of filled composite materials on its basis. Nanosized particles may be used as such fillers [3,4]. The use of such objects to create polymer composites allows to create materials with new and improved properties. Polymer composites containing magnetic nanoparticles are of special interest for the development of materials and coatings on their basis for electromagnetic compatibility, since such materials can be used to create bicomplex electrodynamic media in which the complex dielectric (ε) and magnetic (µ) permeabilities are different from unity. The reason for this is the synergism between the matrix and the filler. A special place among nanoscale fillers is occupied by metal and oxide nanoparticles. The properties of such nanoparticles have opened up the possibility of their use in technological, biomedical and pharmaceutical applications, in creating electronic devices, gas sensors, drug delivery, magnetic hyperthermia, photocatalysis, wastewater treatment and pollutant removal, as well as in high frequency devices, etc. [5,6,7,8].

Nanosized ferrospinels with the general formula MeFe_2_O_4_, where Me = Ni, Co, Cu, Mn, Zn and other elements, look promising. The structure of such compounds consists of a basic unit cell composed of eight sub-element cells. Each such cell has a face-centred cubic spinel structure with tetrahedral (O_A_) and octahedral (O_B_) areas [9]. Depending on the position of the metal cations Me^2+^ and Fe^3+^, a distinction is made between normal, inverse and mixed spinel. The position of Me^2+^ in tetrahedral sites and Fe^3+^ in octahedral sites is characteristic of normal spinel. In the case of inverse spinel, Me^2+^ cations are located in octahedral sites, while Fe^3+^ cations are equally located in both tetrahedral and octahedral sites. Mixed spinels are characterized by the arrangement of Me^2+^ and Fe^3+^ cations equally at both sites. Such nanoparticles have been demonstrated by researchers to possess high antimicrobial properties, biocompatibility, structural stability, magnetic properties, chemical reactivity, adsorption and high catalytic ability [10,11,12,13,14,15,16]. Among the nanoscale ferrospinels, the reverse-structured nanoferrites hold a special place due to their unique magnetic properties and high saturation magnetisation [17].

Nowadays, the nanosized nickel ferrite NiFe_2_O_4_ (NNF) [18] has attracted scientific attention. These nanoparticles exhibit significant magnetoanisotropy due to the arrangement of cations according to the inverse spinel structure. The magnetic characteristics of NNFs differ significantly from microparticles [19,20]. On the one hand, nanosized NiFe_2_O_4_ has a small magnetization and on the other hand, it has a higher coercive force compared to micro-sized nickel ferrite [21]. Nanoscale nickel ferrite exhibits magnetically soft properties as well as magnetic permeability, high electrochemical stability, and catalytic properties as demonstrated in [22,23]. Depending on the particle size and shape, nickel ferrite may exhibit paramagnetic, superparamagnetic and ferrimagnetic properties, low eddy current losses and high electrical resistivity [9,24]. The synergism of these properties in the nanoscale range allows the use of NNFs as catalysts in multicomponent syntheses of organic compounds [25], as active components of gas sensors [26], in magnetic data storage systems, and as an adsorbent for heavy metal removal [27]. NiFe_2_O_4_-based ferrites have also found application in instruments and devices operating in the millimeter and short-wave part of the centimeter wavelength range.

The ability to vary the above properties allows the creation of new composite materials based on nickel nanoferrites with different magnetic characteristics. Composites with NiFe_2_O_4_ nanoparticles are used in electronic devices [28], for example, in [29] it was shown that the use of nanostructured NiFe_2_O_4_ electrodes instead of carbon electrodes can increase the capacity of lithium batteries by about three times.

In this paper the structure of synthesized nickel ferrite nanoparticles contained in high-pressure polyethylene (HPPE) matrix are studied through Mössbauer spectroscopy, XANES and EXAFS methods. The electrical and magnetic properties of these NNFs composites are also investigated. The data obtained indicate interesting properties of nickel-based nanocomposites, which will be useful for obtaining products based on these materials.

## 2. Materials and Methods

### 2.1. Materials

Acetic (glacial acetic) (CH_3_COOH, 99.5%), anhydrous ferric acetate (Fe(CH_3_COO)_3_) and nickel acetate (Ni(CH_3_COO)_2_) were purchased from Acros Organics (Geel, Belgium) and used without further purification. Commercially available high-pressure polyethylene (HPPE) and mineral oil were used.

### 2.2. Preparation of Precursor and Composites

Composites containing nickel ferrite nanoparticles in high-pressure polyethylene (HPPE) were synthesized at 280–300 °C by the decomposition of basic iron (III) acetate and nickel acetate (in mole ratio 1:1) in HPPE melt in the required volume of purified vacuum oil (the volume depends on the size of the flask and the mass of the polymer). The calculated amounts of iron (III) acetate and nickel acetate dissolved in acetic acid were injected into the melt of HPPE in oil under intensive stirring. The synthesis was carried out in an argon atmosphere, which was fed into the reactor, allowing fast and complete removal of gaseous products of salt decomposition and solvent from the reactor. The resulting material was placed in a Soxlet extractor for washing the oil with hexane from the resulting composition. After complete extraction of the oil, the powdered sample was dried. The synthesized composites presented as dark grey powders. Composites with different nickel ferrite contents of 10, 20 and 30 wt.% in the HPPE matrix were obtained. We denote the investigated samples as 10%NiFe_2_O_4_@HPPE, 20%NiFe_2_O_4_@HPPE, 30%NiFe_2_O_4_@HPPE.

### 2.3. Equipment and Measurement

The dimensions of the nanoparticles were determined by transmission electron microscopy using a JEOL JEM-1011 microscope, at an accelerating voltage of 100 kV. The sample under study was subjected to ultrasonic dispersion in hexane, the resulting dispersion was applied to a copper mesh coated successively with layers of formvar and carbon [30,31].

X-ray diffractograms of the studied composites were obtained on diffractometer DRON-3 using CuKα-radiation (λ = 1.54056 Å), which was separated from the general spectrum of the sharp-focus X-ray tube Cu BSV21 using Ni-filter. The diffractograms were recorded in the range of 2θ angles from 5° to 60° with 0.02° step and exposure at the point of 4 sec. Structural parameters of the obtained samples were determined using FullProf Suite software (Version: 2.00) [30,31].

The Mössbauer spectra of the composites, both at room temperature and at low temperature, were measured using the MS1104Em spectrometer. A ^57^Co in the Rh matrix was used as the γ-quantum source. For cooling the samples were placed in a CCS-850 helium cryostat chamber. Model interpretation of the spectra was performed using the SpectRelax software [32]. Isomeric shifts were calculated relative to metallic α-Fe.

EXAFS Ni K- and Fe K-edges of the composites were obtained in the Structural Materials Science beamline of the Kurchatov Center for Synchrotron Radiation of the National Research Center “Kurchatov Institute” (Moscow). X-rays were generated by a storage ring at 2.5 GeV electron beam energy and 100–120 mA average current and decomposed into a spectrum by a Si (111) double-crystal monochromator. The intensities of incident and passed through sample X-rays were recorded by ionization chambers filled with argon. After standard procedures of background extraction, K-edge jump normalization and atomic absorption extraction µ_0_ [33], a Fourier transform of the obtained EXAFS (χ)-spectra in the photoelectron wave vector range k from 2.3 to 13 Å^−1^ with weight function k^3^ was performed. The threshold ionization energy E_0_ was chosen from the value of the maximum of the first derivative of the K-edge and further varied during fitting. Values of local environment parameters of Ni, Fe atoms were determined by approximation of calculated EXAFS to experimental one while varying parameters of corresponding coordination spheres (CS) using IFFEFIT program [34]. The phases and amplitudes of photoelectron wave scattering necessary for model spectrum construction were calculated using FEFF7 program [35]. Structurally characterized metal oxides from the international structural database ISCD were taken as model compounds.

The specific volume resistance ρ_V_ at DC and the dielectric permittivity ε of the composite samples at frequencies 1 kHz and 1 MHz were investigated in a capacitance cell with flat round-shaped electrodes. At measurements an electrometric voltmeter V7E-42 and calibrator P320 as a voltage source, LCR-meters E7–8 (at frequency 1 kHz) and E7–12 (at frequency 1 MHz) were used. Investigations at frequency 1 GHz were performed in the coaxial resonator with an end capacitive gap, Rohde & Schwartz FSP7 spectrum analyzer was used as a signal generator and a resonance indicator. Magnetization curves of the samples, representing disks with diameter of 5 mm and thickness of 1.5 mm, were obtained with automatic vibration magnetometer VM-2K.

Measurements of reflection coefficients R and attenuation factor A (dB/cm) of microwave radiation power were made at 30 GHz using a standing wave ratio (SWR) and attenuation meter R2-65. The samples were investigated in the form of 1.5 mm thick tablets.

## 3. Results and Discussion

**Figure 1:**
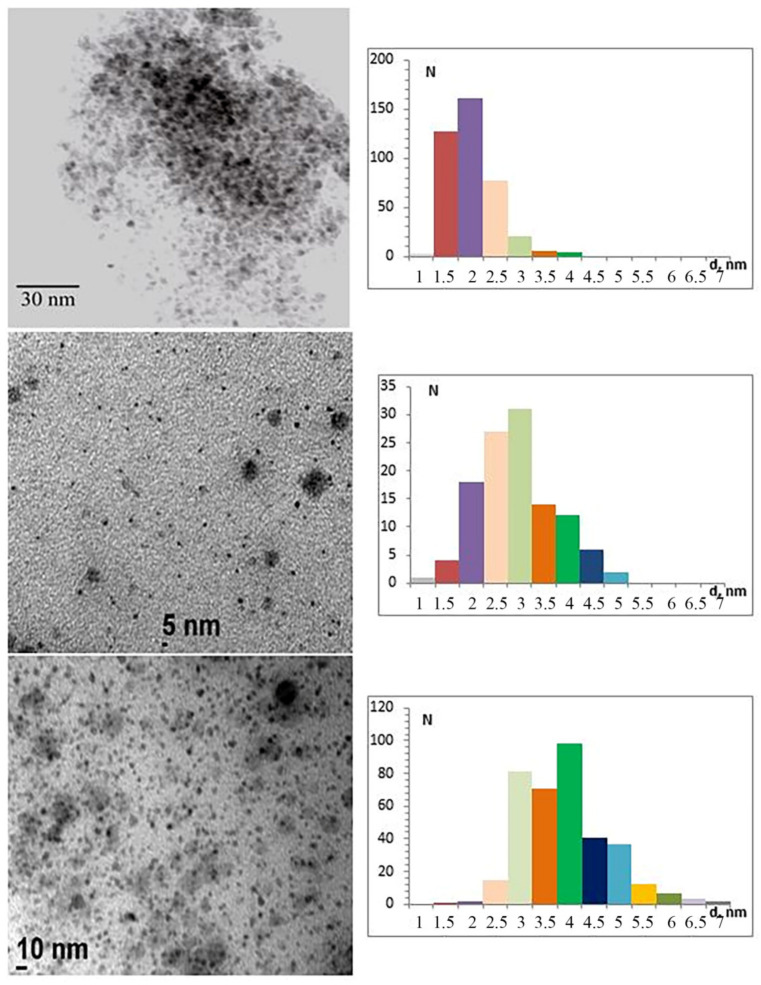
Microphotographs and size distribution diagrams: 1—10%NiFe_2_O_4_@HPPE; 2—20%NiFe_2_O_4_@HPPE; 3—30%NiFe_2_O_4_@HPPE.

The dimensions of the nickel ferrite nanoparticles were determined by transmission electron microscopy (TEM). Figure 1 shows TEM micrographs for the three studied nanocomposite samples.

The size distribution diagrams show that as the metal content of the HPPE matrix increases from 10% to 20% and to 30% while there is an increase in the average size of nanoparticles from 2 to 3 and 4 nm, respectively. The particle size distributions have wide ranges of values, except for the 10%NiFe_2_O_4_@HPPE composite, where 92% of the nanoparticles are in a small size range of 1.5 to 2.5 nm. For the 20%NiFe_2_O_4_@HPPE and 30%NiFe_2_O_4_@HPPE composites, the size interval for the same 92% nanoparticles is in the bracket of 1.5 to 4.0 nm and 2.5 to 5.5 nm, respectively. In each of the composites studied, small amounts of small-size nanoparticles up to 1.5 nm and bigger nanoparticles of 4 nm and larger are detected. In the 10%NiFe_2_O_4_@HPPE composite, it is 2.5% of nanoparticles as large as 3.5 and 4.0 nm. In the 20%NiFe_2_O_4_@HPPE composite, it is about 6.5% nanoparticles of size 4.5–5.0 nm, and in the 30%NiFe_2_O_4_@HPPE composite, it is about 6.5% nanoparticles of size 5.5–7.0 nm.

**Figure 2:**
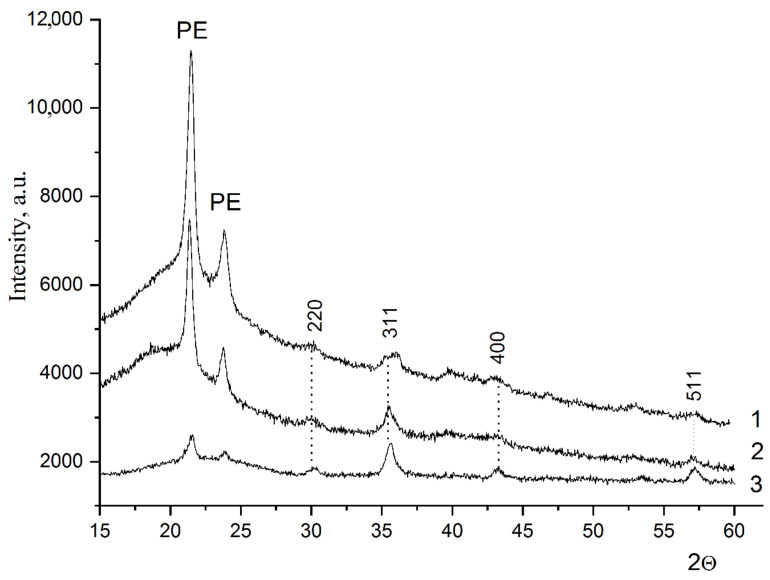
X-ray diffractograms of composites 10%NiFe_2_O_4_@HPPE (1), 20%NiFe_2_O_4_@HPPE (2) and 30%NiFe_2_O_4_@HPPE (3).

The diffractograms of the composites are shown in Figure 2.

The diffractograms show two intense reflexes related to the polyethylene (PE) matrix and four low-intensity broad reflexes with hkl parameters (220), (311), (400), (511) related to the metal-containing filler. The degree of crystallinity of these composites reaches values of about 60–70%. The observed low reflection intensities and their broadening are consistent with the small particle size of the metal-containing component. The decrease in the reflection intensity of the PE matrix with increasing nickel ferrite concentration in the composite indicates that the crystalline phase of HPPE may be destroyed by the filler.

The presence of diffraction reflexes (220), (311), (400), (511) proves the formation of NiFe_2_O_4_ in the composite in cubic syngony (space group Fd-3m, a = 8.338 Å) with the value of the unit cell parameter close to the structure of NiFe_2_O_4_ spinel (ICSD #00-086-2267, a = 8.337 Å). The lattice parameters of the sample determined from the X-ray data are shown in Table 1.

The results obtained for the lattice parameters correlate well with the well-known published data for polycrystalline NiFe_2_O_4_ [36].

The diffractograms (Figure 2) also show an increase in the intensity and decrease in the width of the diffraction reflexes which are typical of NiFe_2_O_4_ as its concentration in the composite grows, which is probably due to an increase of the crystalline phase ratio in NiFe_2_O_4_. In other words, the sizes of nanoparticles become larger during the formation of a crystal structure in more highly filled compositions, which is associated with a longer synthesis.

From the broadening of the diffraction peaks the size of the coherent scattering region was determined, which allowed the estimation of the size of nickel ferrite nanoparticles using the Selyakov-Scherrer formula:

D = nλ/β cosθ (1)

where D is the size of the coherent scattering region in Å, λ is the radiation wavelength, θ is the scattering angle, β is the physical line broadening on the diffractogram in radians, and n is a factor close to 1.

Calculated nanoparticle diameter from reflection parameters (220) and (311) showed that for 10 wt.% nickel ferrite concentration in the composite it was 7 nm, 20 wt.%—10 nm, 30 wt.%—13 nm.

The nanoparticle sizes calculated by the Selyakov-Scherrer formula turned out to be considerably larger than those obtained from the TEM data. Such a discrepancy in the size determination can be explained, primarily, by a wide range of nanoparticle sizes in the composites, which was confirmed by the TEM results, as well as by the accuracy of broadening determination diffraction reflexes. However, the size determined from the Debye−Scherrer expression is always larger than those by TEM. These differences are due to the mode in the size determination:  by X-ray diffraction and TEM, it is assumed to be a volume and a radius distribution, respectively [37,38]. Obviously, the diffraction patterns are characteristic of the crystalline phase, in which larger nanoparticles are formed.

It also should be mentioned that the dependence of the particle size increase on the concentration of the metal-containing phase in the composite is observed by both of the investigation methods.

**Figure 3:**
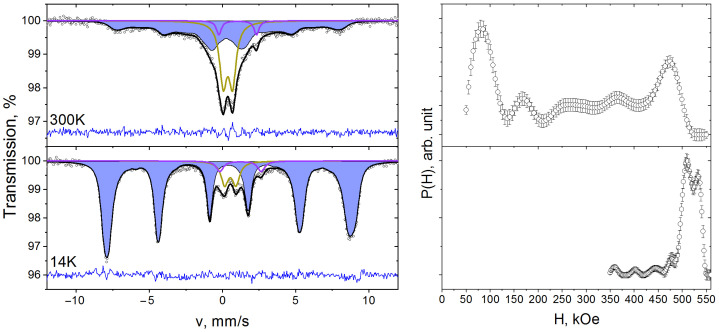
Mössbauer spectra of 10%NiFe_2_O_4_@HPPE sample taken at room temperature and 14 K and restored distribution functions of hyperfine magnetic field P(H).

**Figure 4:**
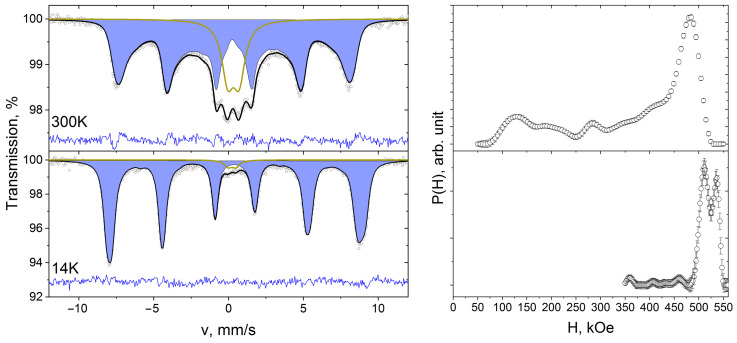
Mössbauer spectra of 20%NiFe_2_O_4_@HPPE sample taken at room temperature and 14 K and restored distribution functions of hyperfine magnetic field P(H).

**Figure 5:**
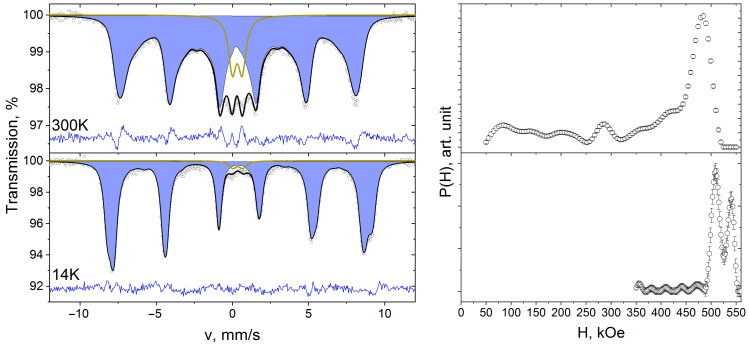
Mössbauer spectra of 30%NiFe_2_O_4_@HPPE sample taken at room temperature and 14 K and restored distribution functions of hyperfine magnetic field P(H).

Mössbauer spectra of synthesized composites were obtained at temperatures of 300 and 14 K (Figure 3, Figure 4 and Figure 5).

At room temperature the spectra consist of quadrupole splitting lines in the center of the spectrum and broadened Zeeman splitting lines. The simultaneous presence of paramagnetic and Zeeman splitting lines in a single Mössbauer spectrum may indicate superparamagnetic properties of the composites under study [39]. The doublet component corresponds to Fe^3+^ ions in nanoparticles with a magnetic moment rotation frequency greater than the Larmor precession frequency of ^57^Fe nuclei [40]. Since the value of the superparamagnetic relaxation frequency is greater the smaller the size of the nanoparticles, the doublet component corresponds to small nanoparticles. The Zeeman splitting lines arise from larger nanoparticles whose rotational frequency of magnetic moment is less than or equal to the Larmor precession frequency. The broadening of the Zeeman splitting lines is probably due to the particle size distribution.

To describe the Mössbauer spectra, the doublet components and the superfine magnetic field distribution function P(H) were used. The values of isomer shift δ and quadrupole splitting Δ of doublet D spectra of 10%NiFe_2_O_4_@HPPE, 20%NiFe_2_O_4_@HPPE, 30%NiFe_2_O_4_@HPPE at room temperature are ≈0.34 mm/s and ≈0.70 mm/s, respectively. The values of the doublet D areas for the samples of the above composites are 32%, 19% and 10%. Note that the spectrum of the 10%NiFe_2_O_4_@HPPE composite exhibits a second doublet of DP, with δ ≈ 1.04 mm/s and quadrupole Δ ≈ 2.58 mm/s, which most probably corresponds to the Fe^2+^ ions of the impurity phase.

The superfine magnetic field distribution functions P(H) are in the range of H values from 50 kE to 500 kE and consist of a set of local maxima that correspond to the Fe^3+^ ions in nanoparticles of different sizes. The fact that the dependence of the P(H) function on the nanoparticle size is also indicated by a shift of the most intensive maxima of the P(H) function towards higher H values with increasing nickel ferrite content in the HPPE matrix, which agrees well with TEM data (Figure 1) on the increase in nanoparticle size in the series composites 10%NiFe_2_O_4_@HPPE, 20%NiFe_2_O_4_@HPPE, 30%NiFe_2_O_4_@HPPE.

Reduction of temperature leads to the decrease of nanoparticles’ superparamagnetic relaxation frequency, and the doublet component area in Mössbauer spectra (Figure 3, Figure 4 and Figure 5) decreases too [40]. At 14 K, the area of doublets in the composite spectra decreases to 7% in the case of 10%NiFe_2_O_4_@HPPE sample and to 2% for 20%NiFe_2_O_4_@HPPE, 30%NiFe_2_O_4_@HPPE, which agrees with TEM data, according to which the number of small size nanoparticles decreases with increasing filler concentration. The P(H) functions recovered for these spectra consist of two intense maxima in the vicinity of 510 and 530 kE and a set of local maxima of low intensity in the H range from 350 to 500 kE.

**Figure 6:**
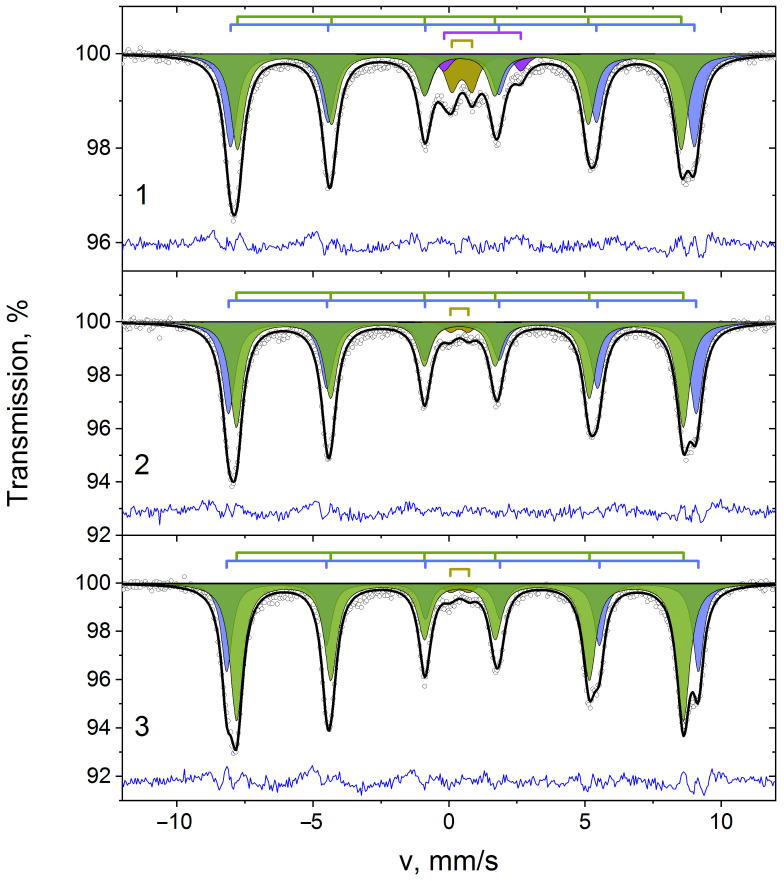
Mössbauer spectra of composite samples taken at 14 K for 10%NiFe_2_O_4_@HPPE (1), 20%NiFe_2_O_4_@HPPE (2), 30%NiFe_2_O_4_@HPPE (3).

Figure 6 shows the spectra of material samples measured at 14 K and decomposed into sets of two sextets and doublets. As can be seen, the spectra of all composites (Figure 6) are made up of a doublet corresponding to small particles with unlocked magnetic moment and two Zeeman sextets.

The Mössbauer spectrum of 10%NiFe_2_O_4_@HPPE contains the additional doublet DP, which corresponds to the impurity phase. The hyperfine parameters of Mössbauer spectra taken at 14 K for nanocomposites samples are listed in Table 2. The δ values of SA и SB sextets is 0.40 mm/s and 0.50 mm/s and typical for Fe^3+^ in oxygen tetrahedron and octahedron respectively.

The SA sextet corresponds to the Fe^3+^ ions occupying the A-sublattice of NiFe_2_O_4_, while the SB sextet refers to Fe^3+^ ions in the B-sublattice [41]. The SA and SB sextet areas (A) are approximately proportional to the Fe^3+^ ion concentrations in the corresponding tetrahedral and octahedral oxygen environments.

In the material 10%NiFe_2_O_4_@HPPE the A areas of SA and SB sextets are approximately equal (see Table 2). With increasing average size of nanoparticles, an increase of SA sextet area of 52% for 20%NiFe_2_O_4_@HPPE composite and 64% for 30%NiFe_2_O_4_@HPPE composite is observed.

Thus, as the nickel ferrite content in the HPPE matrix increases from 10 to 20 and 30 wt.%, the iron ion phase increases from 45% to 52% and 64% in the tetrahedral environment.

The local environments of iron and nickel atoms in 10%NiFe_2_O_4_@HPPE, 20%NiFe_2_O_4_@HPPE and 30%NiFe_2_O_4_@HPPE nanoparticles were studied by X-ray absorption spectroscopy.

**Figure 7:**
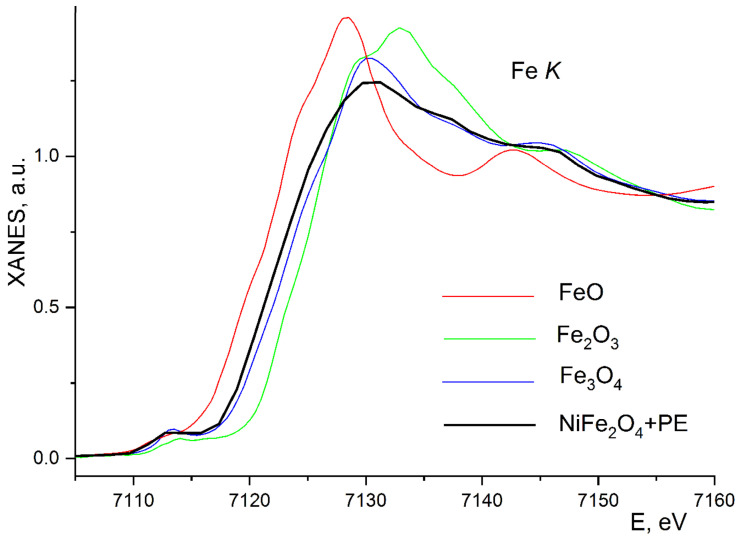
Normalized XANES Fe K absorption edges for 10%NiFe_2_O_4_@HPPE composite and FeO, Fe_2_O_3_, Fe_3_O_4_ standards.

The degree of oxidation of iron ions in nanoparticles of the 10%NiFe_2_O_4_@HPPE composite was clarified by the energy position of the X-ray K-edge of iron absorption compared with their values for the compounds-standards. Figure 7 shows the Fe K-edge XANES for 10%NiFe_2_O_4_@HPPE and compounds of standards FeO, Fe_3_O_4_, Fe_2_O_3_.

The X-ray absorption edges energy position has practically linear dependence on the oxidation degree of the absorbing iron ion, shifting towards higher energies with its increase. When the oxidation degree changes from 2+ (FeO) to 2.67+ (Fe_3_O_4_) and 3+ (Fe_2_O_3_), the edge shift is 0, 4.6 and 7.2 eV, respectively (Figure 7). In approximation of linear dependence, the average oxidation degree of Fe ion in 10%NiFe_2_O_4_@HPPE material can be estimated, and it turned out to be approximately equal to 2.5+. This value of the oxidation degree of Fe ions in the composite agrees very well with the data of Mössbauer spectroscopy.

The position of Ni K absorption edges for all three composites coincides well with the edge position for NiO, indicating that the Ni ions in nanoparticles have an oxidation degree of 2+.

The parameters of the nearest atomic environment of iron and nickel ions in nanoparticles of the studied materials were determined from XANES and EXAFS analysis of Fe K- and Ni K- absorption edges.

**Figure 8:**
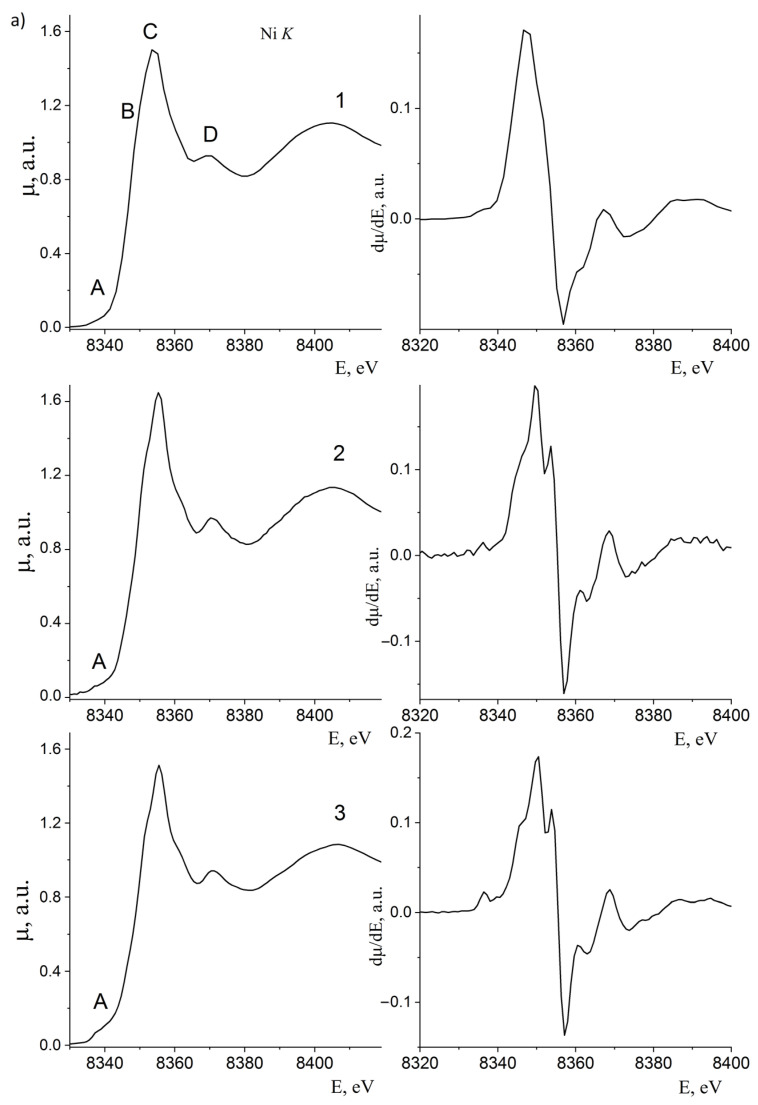

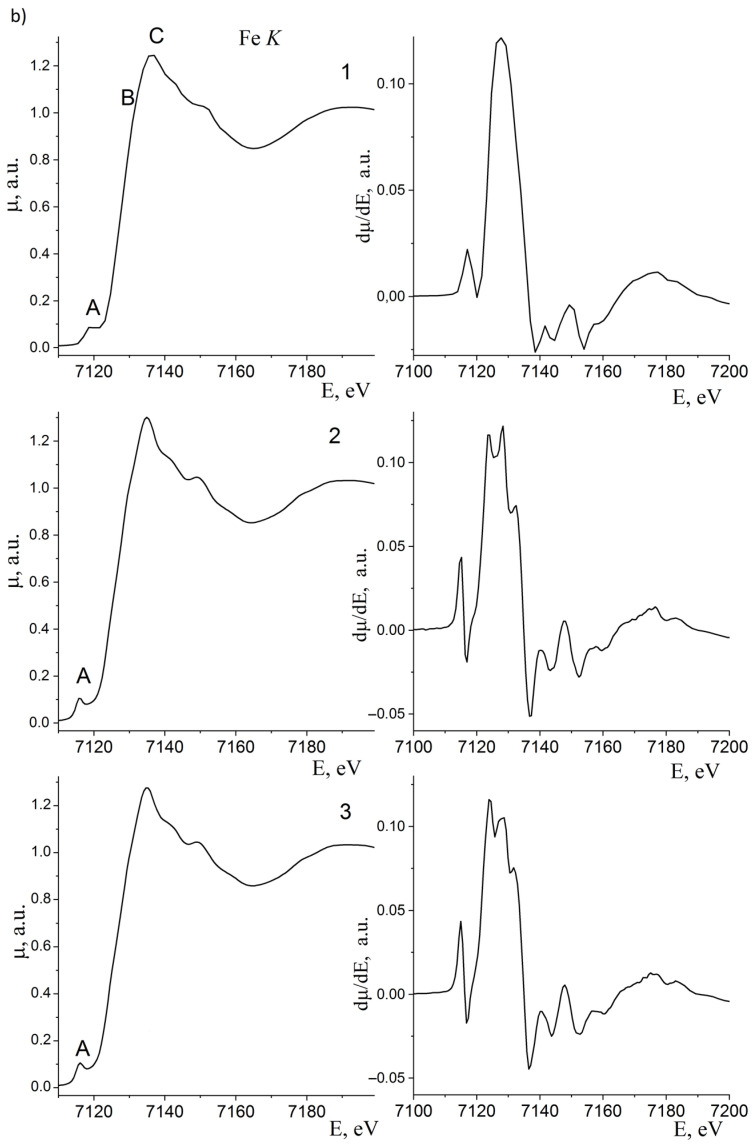
(**a**) Normalized XANES Ni K-edges (first derivatives shown on the right). 1—10%NiFe_2_O_4_@HPPE; 2—20%NiFe_2_O_4_@HPPE; 3—30%NiFe_2_O_4_@HPPE. (**b**) Normalized XANES Fe K-edges (first derivatives shown on the right). 1—10%NiFe_2_O_4_@HPPE; 2—20%NiFe_2_O_4_@HPPE; 3—30%NiFe_2_O_4_@HPPE.

Figure 8 shows normalized XANES of Ni K- and Fe K- absorption edges and their first derivatives dμ/dE.

The fine structure of XANES depends on the symmetry of the surrounding of the absorbing ion. Pre-edge A peaks can appear only in the absence of a symmetrical environment of metal ions due to the mixing of p-d AO.

As can be seen from Figure 8, the pre-edge structure at the Ni K edge, peak A, is practically absent, indicating a symmetrical environment for nickel ions.

However, the first derivatives dμ/dE of these K-edges show a split maximum indicating a distorted octahedral environment of nickel ions.

In contrast to the Ni K-edge, the Fe K-edges, Figure 8, show an intense pre-edge A structure and the first derivative dμ/dE is broadened and has several well-defined maxima. Therefore, the iron ion in the nanoparticles has a low symmetry environment. Given the PCA findings on the crystal structure of nanoparticles, this is due to different octahedral and tetrahedral iron ion environment symmetries.

**Figure 9:**
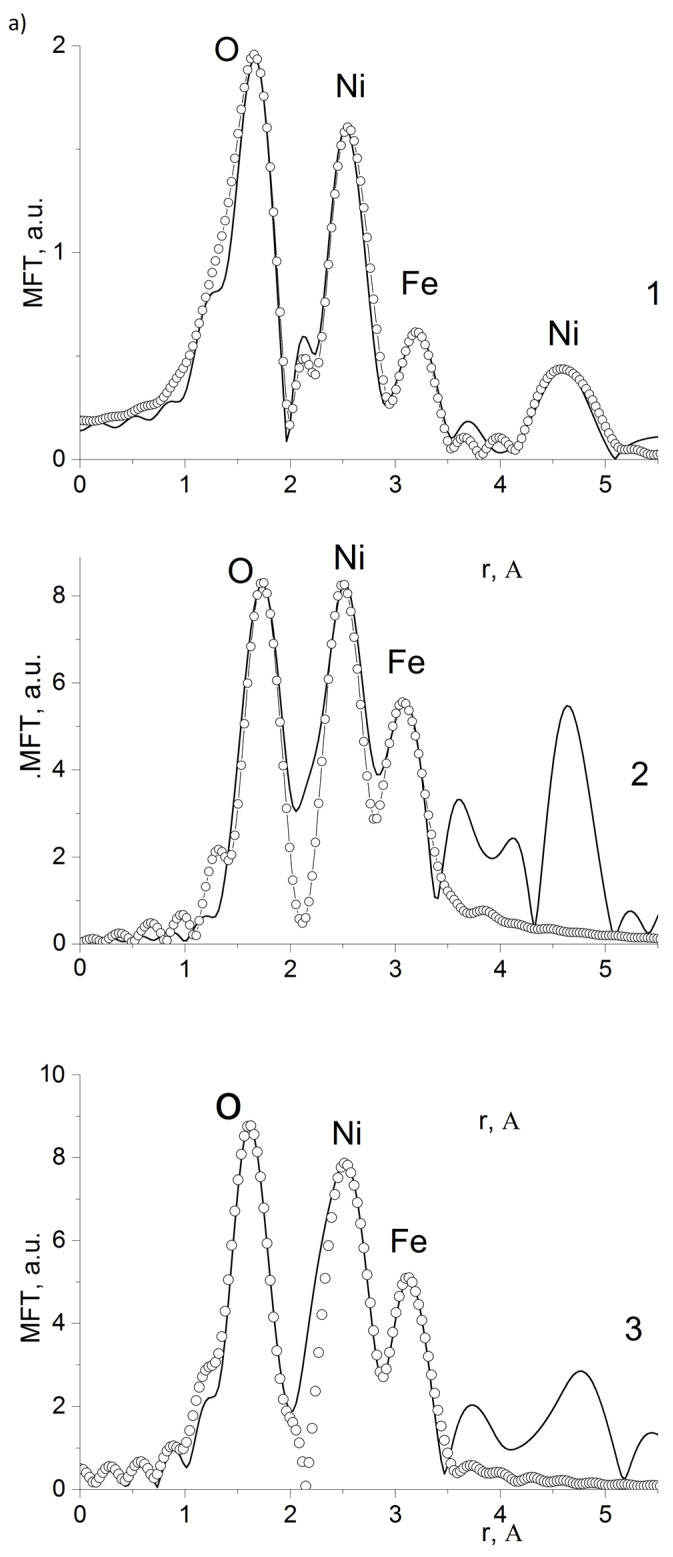

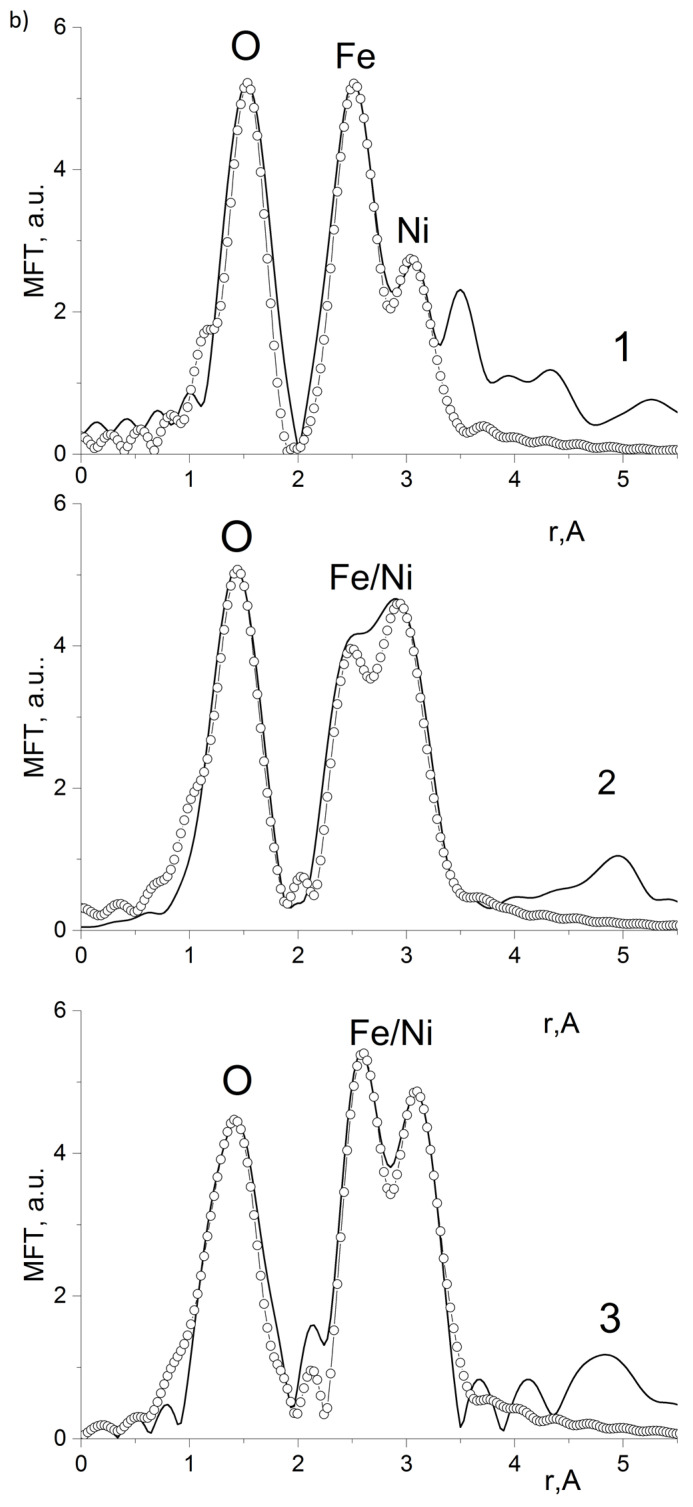
(**a**) FTM EXAFS Ni K-edges; (solid line—experiment, empty circles—calculation) 1—10% NiFe_2_O_4_@HPPE; 2—20%NiFe_2_O_4_@HPPE; 3—30%NiFe_2_O_4_@HPPE. (**b**) FTM EXAFS Fe K-edges; (solid line—experiment, empty circles—calculation) 1—10% NiFe_2_O_4_@HPPE; 2—20%NiFe_2_O_4_@HPPE; 3—30%NiFe_2_O_4_@HPPE.

The parameters of the local atomic structure of metal ions in nanoparticles of composites are determined from the EXAFS analysis of Ni K- and Fe K-absorption edges of nickel ferrite nanoparticles in composites whose Fourier transform modulus (FTM) is given in Figure 9.

A model of metal ions local atomic structure in nanoparticles has been chosen based on the crystal structure of NiFe_2_O_4_ ferrite. In the FTM of Ni K- and Fe K- edges the first peaks at r = 1.66 Å and 1.54 Å, respectively, are due to the manifestation of the first edge structure of the oxygen environment around nickel and iron ions. For iron ions, we used a two-sphere model (tetrahedral and octahedral environments of iron ions) in the fitting. When using a single-sphere model, the discrepancy function Q of the EXAFS approximation was 2.5 times worse. Table 3 shows the structural data of the nearest atomic environment of Fe and Ni ions obtained from the multi-sphere EXAFS fitting for the investigated composites.

As Table 3 vividly demonstrates there are two average distances of Fe…O in nanoparticles—a short one about 1.85–1.91 Å in tetrahedral and a long one about 2.00–2.05 Å in octahedral environments. The longer mean distances Ni…O of 2.04–2.13 Å correspond well to the values for the octahedral environment of nickel ions. Both for nickel ions and iron ions, reduced coordination number values appear to be due to the size effects of the nanoparticles. For farther Fe…Ni and Ni…Fe the comparable radii (Table 3) are obtained, with a typical parameter determining error for such edge structures of about 0.03 Å. The values of local atomic environment of Fe and Ni ions in NiFe_2_O_4_@HPPE are close to the previously obtained parameters of the local structure in NiFe_2_O_4_ nanoparticles in other matrices.

The manifestation of long-range coordination spheres up to 3–4 Å indicates a high degree of atomic ordering in NiFe_2_O_4_@HPPE nanoparticles and can serve as a confirmation of the good crystallinity of the nanoparticles.

**Figure 10:**
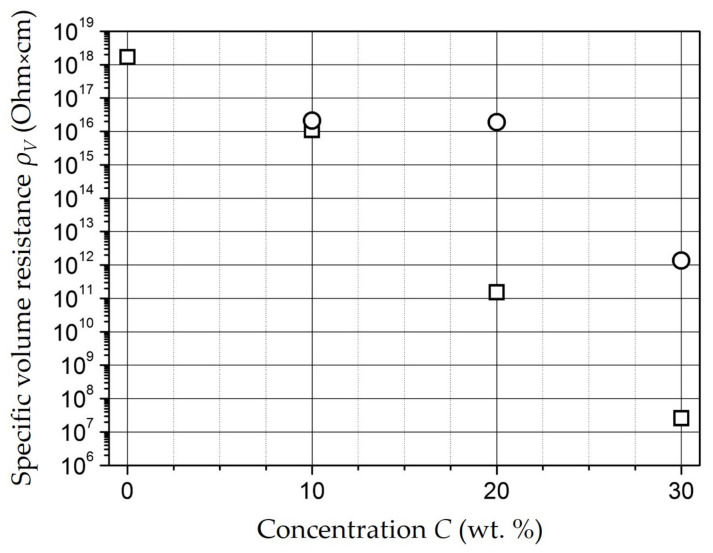
Dependence of ρ_V_ on concentration.

The study of the specific volume resistance (ρ_V_) of the samples showed that ρ_V_ decreases with increasing concentration, but up to the concentration of 20 wt.% it remains comparable to ρ_V_ of unfilled polyethylene—10^16^…10^18^ Ω × cm (Figure 10).

A noticeable decrease in ρ_V_ is observed at a concentration of 30 wt.%—10^12^ Ω × cm for a 1.5 mm thick tablet (circles in Figure 10). In the film samples (squares in Figure 10) an even more marked decrease in conductivity of 10^7^ Ω-cm is achieved in the 30 wt.% concentration sample.

**Figure 11:**
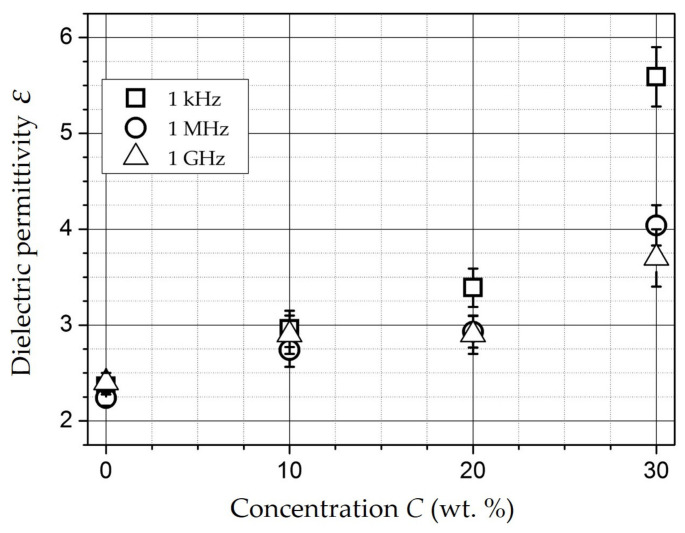
The ε dependence on concentration.

At frequencies of 1 kHz (squares in Figure 11), 1 MHz (circles in Figure 11), and 1 GHz (triangles in Figure 11), ε of samples (1.5 mm thick) also increases with increasing concentration, and relatively lightly changing at C = 5…20 wt.% (ε = 2.7…3.4), reaches values 5.6 (1 kHz), 4 (1 MHz) and 3.7 (1 GHz) at C = 30 wt.%.

**Figure 12:**
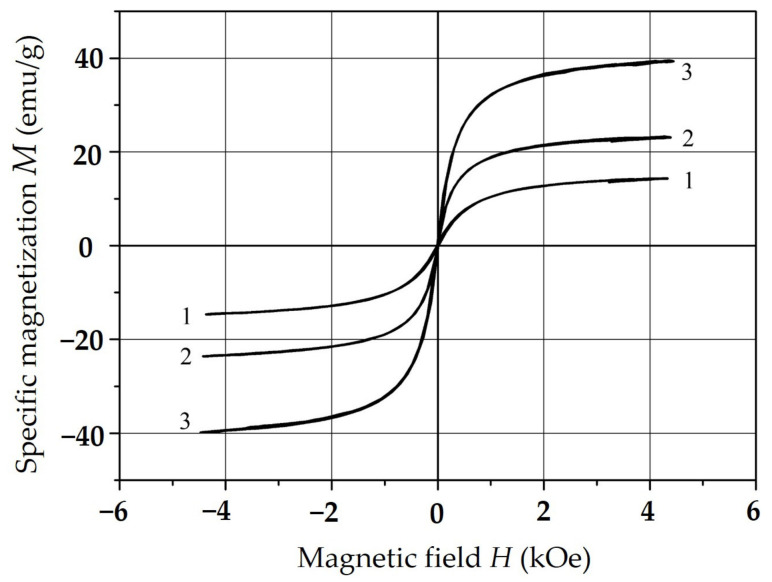
Sample demagnetization curves.

The demagnetization curves of the samples with C = 5 (curve 1), 10 (curve 2), 20 (curve 3) and 30 (curve 4) wt. % are presented in Figure 12.

**Figure 13:**
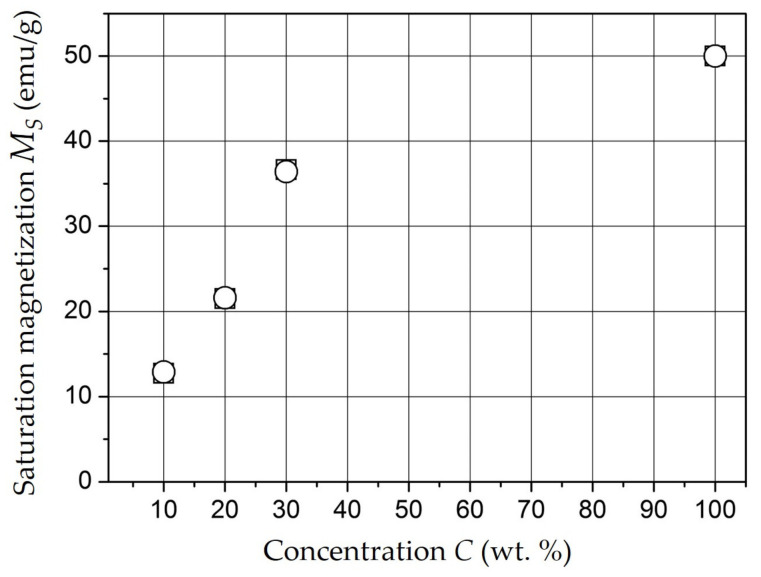
Dependence of M_S_ on concentration C.

In the analysis of these curves, we found that the studied materials can be classified as magnetically soft, the saturation magnetization M_S_ in them is achieved in the fields of 2 kOe and increases with increasing C concentration. Dependence of M_S_ (emu/g) on C is shown in Figure 13. Point C = 100 wt.% is corresponded to a tabular value for massive NiFe_2_O_4_.

**Figure 14:**
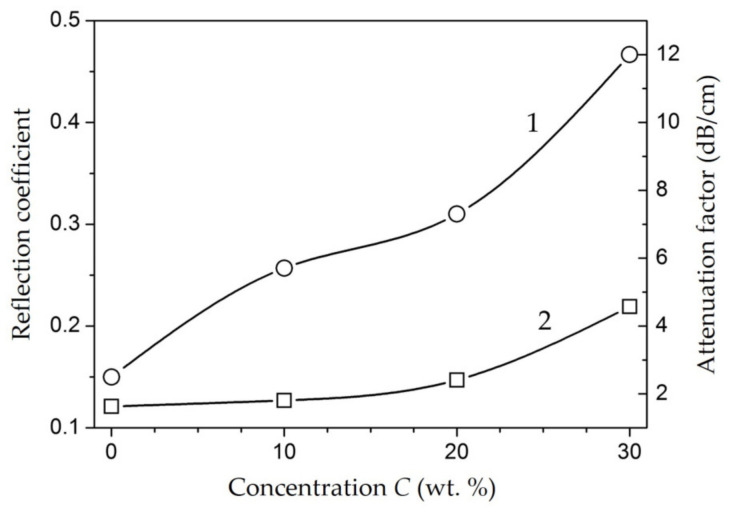
The reflection coefficient and attenuation factors dependence on concentration.

The measurement of the reflection coefficients (R) and attenuation factor (A) of the microwave power of the composite samples demonstrated that R and A increase with growing concentration (Figure 14). Notice that up to C = 20 wt.% A value is in the range of 5…7 dB/cm with relatively low R (0.13…0.15), which is slightly higher than for unfilled HPPE (2.5 dB/cm and 0.12). The major increase in A and R values is observed in the concentration range of 20…30 wt.% (up to 12 dB/cm and 0.22).

## 4. Conclusions

NiFe_2_O_4_ nanoparticles in a high-pressure polyethylene matrix with concentrations of 10 wt.%, 20 wt.% and 30 wt.% have the structure of a cubic spinel (Fd-3m, a = 8.338 Å). As the concentration of NiFe_2_O_4_ nanoparticles increases from 10 wt.% to 20 wt.% and to 30 wt.% in polyethylene, their average sizes increase from 2 to 3 and to 4 nm, respectively. The size distributions for nanoparticles of each of the composites have rather wide value intervals, from 1.5 to 4 nm for the 10%NiFe_2_O_4_@HPPE composite, from 1.5 to 5 nm for 20%NiFe_2_O_4_@HPPE and from 2.5 to 7 nm for 30%NiFe_2_O_4_@HPPE.

Mössbauer studies have shown that with increasing NiFe_2_O_4_ concentration in polyethylene, magnetic ordering of iron ions occurs due to the increase in nanoparticle size. The spectra at 15K were doublets (δ = 0.42 ± 0.03 mm/s and Δ = 0.75 ± 0.07 mm/s), and two sextets with H1 = 507.5 ± 1.5 kE and H2 = 534 ± 4 kE corresponding to the tetrahedral and octahedral positions of iron in crystalline NiFe_2_O_4_. As the nickel ferrite content in the HPPE matrix increases from 10 to 20 and to 30 wt.%, the iron ions phase increases from 45 to 52 and 64 wt.% in the tetrahedral environment.

The Ni-O distances in the octahedron are R= 2.08 Å (N = 5.4). Fe-O distances in the tetrahedron are R= 1.88 Å (N = 1.5), Fe-O distances in the octahedron are R= 2.03 Å (N = 1.5). Comparable Ni-Fe radii were obtained for farther Fe…Ni or Ni…Fe spheres with R = 3.43 ± 0.03 Å.

We obtained magnetically soft magnetodielectric materials based on NiFe_2_O_4_ nanoparticles and HPPE matrix and their saturated magnetization depends on the nanoparticles concentration and is comparable in magnitude with the saturated magnetization of solid nickel ferrite.

## Figures and Tables

**Table 1:** Crystal structure parameters of NiFe_2_O_4_ samples obtained from the experimental data processing.

Composition	NiFe_2_O_4_
Spatial group	Fd-3m
Syngony	cubic
Parameters, a, Å	8.338
Bragg factor	2.73

**Table 2:** The parameters of the Mössbauer spectra at 14 K for the composite 10%NiFe_2_O_4_@HPPE, 20%NiFe_2_O_4_@HPPE, 30%NiFe_2_O_4_@HPPE—Samples 1, 2 and 3, respectively.

Samples	Component	δ ± 0.02, mm/s	Δ/ε ± 0.02, mm/s	H ± 1, kOe	Γ ± 0.02, mm/s	A ± 1, %
1	D	0.45	0.82		0.60	7
	DP	1.23	2.80		0.60	4
	SA	0.40	−0.01	506	0.53	45
	SB	0.50	0.01	530	0.53	44
2	D	0.39	0.66		0.54	2
	SA	0.40	−0.01	509	0.58	52
	SB	0.49	0.01	533	0.58	46
3	D	0.40	0.68		0.57	2
	SA	0.40	−0.01	509	0.48	64
	SB	0.50	−0.01	538	0.48	34

δ—isomer shift, ε—quadrupole shift, Δ—quadrupole splitting for paramagnetic component, H—hyperfine magnetic field on ^57^Fe nucleus, Γ—linewidth, A—component area.

**Table 3:** Structural data of the closest atomic environment of Fe and Ni ions in NiFe_2_O_4_@HPPE obtained from EXAFS multisphere fitting (N—coordination numbers, R—interatomic distances, 2—Debye-Waller factor, Q—non-coupling function).

Edge and Composite	N	R, Ǻ	σ2, Ǻ2	Edge Structure	Q, %
Fe K-edge 10%NiFe_2_O_4_@HPPE	1.5	1.91	0.0035	O (tetra)	4.0 *
	1.5	2.05	0.0035	O (octahedron)	
	1.6	2.92	0.0045	Fe	
	1.3	3.39	0.0045	Ni	
Ni K-edge 10%NiFe_2_O_4_@HPPE	5.4	2.08	0.0040	O (octahedron)	6.4 **
	4.2	2.97	0.0050	Ni	
	3.2	3.41	0.0050	Fe	
Fe K-edge 20%NiFe_2_O_4_@HPPE	1.6	1.89	0.0035	O (tetra)	7.8 **
	1.5	2.01	0.0035	O (octahedron)	
	1.6	2.98	0.0045	Fe	
	3.3	3.44	0.0045	Ni	
Ni K-edge 20%NiFe_2_O_4_@HPPE	3.4	**2.13**	0.0035	O (octahedron)	3.5 **
	2.9	2.91	0.0050	Ni	
	4.1	3.42	0.0050	Fe	
Fe K-edge 30%NiFe_2_O_4_@HPPE	1.6	1.85	0.0035	O (tetra)	8.6 **
	1.5	2.00	0.0035	O (octahedron)	
	2.1	3.02	0.0045	Fe	
	3.3	3.43	0.0045	Ni	
Ni K-edge 10%NiFe_2_O_4_@HPPE	4.0	2.04	0.0035	O (octahedron)	9.9 **
	2.9	2.92	0.0050	Ni	
	3.0	3.46	0.0050	Fe	

* Δr = 1.0–3.3 Ǻ, ** Δr = 1.0–5.0 Ǻ—fitting interval.

## Data Availability

Data available upon request.
